# A User-Centric Approach to the Development of a Mobile Yoga App for Patients With Thoracic Cancer and Their Family Caregivers

**DOI:** 10.2196/89649

**Published:** 2026-10-30

**Authors:** Kathrin Milbury, Olivia Chang, Meagan Whisenant, Jason R Bentley, Scherezade K Mama, Yong Cui, Yisheng Li, Rosangela F Silva, Nita Patel, Sania Yousuf, Aileen Chen, Susan K Peterson

**Affiliations:** 1 The University of Texas MD Anderson Cancer Center Houston, TX United States; 2 The University of Texas Health Science Center at Houston Houston, TX United States; 3 University of Houston - Clear Lake Houston, TX United States

**Keywords:** prototype development, mobile yoga app, yoga, thoracic cancer, caregivers, user-centric approach

## Abstract

**Background:**

We recently completed the efficacy testing of an instructor-led yoga program for patients with thoracic cancer undergoing radiotherapy and their family caregivers. Translating an instructor-led intervention to a mobile app–based delivery model may facilitate the implementation of this yoga program in routine oncological clinical care.

**Objective:**

The current study seeks to develop a prototype of the app-based yoga program using a user-centric approach.

**Methods:**

Ten patients with a primary diagnosis of lung or esophageal cancer and their family caregivers (N=20) participated in structured interviews. Half of the sample had previously participated in the instructor-led yoga program, whereas half of the sample did not. Participants provided feedback on various mock-ups, wireframes, and yoga videos to guide the development of the app’s content, features, and workflow.

**Results:**

All patients (60% male; 70% non–Hispanic White; age: mean 62 years; 90% stage III) and their caregivers (90% female; 50% non–Hispanic White; age: mean 52 years) indicated that developing an app-based program is important and useful. Of the 20 participants, 95% mentioned that having an initial orientation with a yoga interventionist is important, and 90% stated that at least 1 check-in should be available (65% desired weekly check-ins). Participants provided feedback on characteristics of the instructor and instruction preferences. Other desired features included scheduling sessions on a calendar (95%), monitoring progress (95%), receiving practice reminders (90%), providing education on yoga as symptom management (90%), and providing daily inspiration (85%). All participants indicated the importance of including practices of various lengths.

**Conclusions:**

Both patients and caregivers deemed an app-based yoga program useful. Regardless of their level of yoga experience, almost all participants desired at least some contact with a live yoga instructor, although the desired frequency varied. These data have informed the development of a prototype that we are currently field-testing to ensure feasibility and acceptability.

## Introduction

People with thoracic cancers (ie, lung and esophageal cancers) often receive aggressive, multimodal treatment to manage their disease. They commonly experience disease- and treatment-related burdens such as decreased lung function and increased dyspnea, which is a subjective symptom characterized by shortness of breath [1]. Other symptoms, such as fatigue and depressive symptoms, may further contribute to reduced functional performance and compromised overall quality of life (QOL) [2]. In addition, patients with thoracic cancers tend to be older and thus vulnerable to experiencing comorbidities that compound symptom burden.

Family caregivers are important sources of care for patients with cancer. Yet, many caregivers experience their own distress, loss of sleep, and fatigue that impair their ability to provide quality care for their loved ones [3]. These manifestations could be due to physical caregiver burden as well as the emotional toll associated with a caregiving role [4]. As QOL is interdependent in families coping with cancer, meaning that caregivers have an impact on patients’ QOL and vice versa, it is crucial to use a dyadic approach that teaches both patients and caregivers how to manage symptoms together [5]. Studies have demonstrated that this joint effort is more feasible and effective than individual coping strategies [6,7].

Yoga may be an effective symptom management strategy to mitigate cancer-related burden, as it involves physical exercises combined with breathing techniques to relieve stress. Yoga could be particularly relevant and helpful for patients struggling with respiratory distress and declines in functional capacity [8]. We have developed a yoga program seeking to address the needs of patients with thoracic cancers and their family caregivers [9-11]. Although we have shown that the instructor-led program delivered in person at the hospital or via videoconference is feasible, a mobile app may facilitate the implementation of yoga into routine clinical care, particularly for patients undergoing a standard radiation treatment schedule (ie, >20 fractions). A mobile yoga app may provide access to supportive care as it increases flexibility for caregivers’ participation and decreases costs for low-income populations who may experience barriers to receiving supportive care [12-14].

In fact, mobile exercise apps have already been developed for people with lung cancer. For instance, the iEXHALE app aimed to increase exercise and improve symptoms for lung cancer survivors who had completed cancer treatment. Participants valued the exercises for managing lung cancer symptoms as well as the app’s self-management capabilities [15]. Mobile apps also hold promise to deliver cardiopulmonary rehabilitation after surgery for early-stage lung cancer survivors to improve cardiopulmonary fitness, QOL, and symptom burden [16]. Similarly, for patients with advanced lung cancer undergoing chemotherapy, evidence based on 2 single-arm trials suggests that app-based pulmonary rehabilitation programs appear to improve functional capacity and cancer-related symptoms [17,18].

With exercise apps showing potential for improving QOL and physical function for patients with lung cancer (with much less known about patients with esophageal cancer), the development of a yoga app for symptom management seems feasible. Although there is no previous literature targeting patients with lung or esophageal cancer, the development of yoga apps for patients with cancer and specifically patients with breast cancer is underway [19]. For instance, the Mindfulness Breast Care (MBC) app includes content on health education regarding body image and stigma as well as daily Hatha yoga videos for practice. Participants appreciated the app’s functionality, esthetics, information quality, and perceived impact [20,21]. Although users indicated high ratings for the overall MBC app, their experiences with the yoga videos were not separately assessed.

With these considerations in mind, the goal of our study is to inform the development of a prototype of a mobile yoga app. Following a user-centric approach, this study sought to solicit feedback from patient-caregiver dyads on key content, design decisions, and enhancement features of the prototype with the intent to develop a mobile yoga app for future feasibility, acceptability, and efficacy testing.

## Methods

### Participants

Our sample included 10 patient-caregiver dyads (N=20) who had completed participation in a clinical trial (NCT03948100). The parent randomized controlled trial examined the efficacy of an instructor-led yoga intervention compared to a psychoeducation intervention for patient-caregiver dyads [11]. The yoga and education sessions were delivered initially in person at the hospital and then remotely via Zoom (Zoom Communications Inc) after the onset of the COVID-19 pandemic. Eligibility criteria for the parent trial included patients who were diagnosed with non–small cell lung or esophageal cancer, were undergoing at least 4 weeks of radiation therapy, had an Eastern Cooperative Oncology Group (ECOG) performance status of ≤2, and had a family caregiver willing to participate in the study. Both patients and caregivers had to be aged ≥18 years, proficient in written and spoken English, able to provide informed consent, and have access to the internet. Patients who practiced yoga within the year of diagnosis were excluded. The research team had the capability to offer loaner devices; however, all participants had access to a computer, smartphone, or tablet. After participants had completed all trial-related procedures, we invited 5 consecutive dyads from the yoga group and 5 consecutive dyads from the education group to participate in structured interviews. The interviewing of participants from both study conditions served the purpose of including the perspectives from patients and caregivers who had different levels of yoga experience in the development of the prototype for a mobile yoga app.

### Procedures

We recontacted participants who had completed the aforementioned parent trial by telephone, informed them that the purpose of the recontact was to seek their participation in an optional interview to provide feedback on the development of a prototype of a mobile yoga app for patients with thoracic cancers and their family members. After providing verbal informed consent, patients and caregivers completed individual, structured interviews via videoconference (Zoom), which lasted approximately 30 to 45 minutes.

During the interview, participants were asked to describe their current mobile app use. Then, using the shared screen option in Zoom, we presented participants with different mock-ups, wireframes, and yoga video samples to gather their feedback about app content, instruction preferences, and functional features. In addition to open-ended questions (eg, “Describe any suggestions to improve this screen”), we mainly included Likert scale items ranging from “not important” to “very important” to solicit specific feedback on content and features. The interview guide was developed specifically for the purpose of this study to inform design decisions. The mock-ups, along with the interview guide, can be found in Multimedia Appendix 1.

All interview recordings and field notes (capturing the date, time, interviewers, participant, and context) were professionally transcribed. Recordings and transcripts were stored on an institutional server. We used content analysis to analyze the interview transcripts. Participants’ demographic and clinical information was provided during the baseline assessment of the parent trial. The sample size of 10 dyads (ie, 20 individual interviews) was determined based on prior methodological research suggesting that saturation is typically achieved within 12 to 20 interviews in relatively homogeneous samples [22].

### Structured Interviews

Two yoga therapists (RFS and NP) who were trained by a qualitative methods expert (MW) conducted the interviews. Training included the completion of 3 recorded mock interviews that the research team viewed and approved. Following the interview guide (Multimedia Appendix 1), participants were asked general questions assessing their previous experience with using mobile apps, inquiring if they had any apps on their mobile device or tablet, and which apps they used if they answered “yes.” We asked participants to evaluate features such as daily inspirational quotes, scheduling, and progress monitoring features, and the importance of including an education tab that would highlight how the yoga program can be used to manage symptoms (eg, meditations to reduce sleep disturbances, breathing exercises to help with anxiety, and physical exercises to improve fatigue). We devoted a considerable portion of the interviews to learning about participants’ preferences regarding the app-based yoga instructional videos. We played 4 videos (2 that were 90 seconds and 2 that were 40 seconds in length) in a counterbalanced order and asked questions pertaining to the instruction style. We played the shorter videos to learn about participants’ cueing preference. More specifically, we asked if participants preferred voice-over (ie, a narrator delivering the instructions) vs direct cueing (ie, the instructor speaking directly to the user). The longer yoga videos featured either 3 instructors, each demonstrating a different fitness level (ie, chair-based, intermediate standing, or full expression of the pose), or a single instructor demonstrating the yoga postures at a specific fitness level. We asked participants if the yoga videos included in the app should feature the same instructor or if several instructors should be featured and inquired about preferred instructor characteristics (age, race or ethnicity, sex, and body type). We also solicited feedback regarding the importance of contact with the instructor, such as an initial orientation with a live instructor via Zoom or another remote videoconference software and additional touchpoints throughout the program, as well as the importance of obtaining technical support. Participants rated how important it would be to have those features and indicated their preferences as part of the app design. For sex, age, body type, and race or ethnicity, they were asked to indicate the degree of importance of having a representative instructor with these characteristics, using a scale of “Important,” “Not Important,” and “Neutral.”

### Ethical Considerations

The MD Anderson Cancer Center Institutional Review Board (IRB) approved recontacting study participants for the purpose of the current study under a modification for protocol 2018-0503 using verbal informed consent. Participants were informed that the interviews would be audio recorded during the informed consent process, as well as again right before starting the interview. Audio files of the interviews were labeled using study IDs rather than identifying information (eg, medical record numbers or participants’ names). An institutionally vetted transcription service that was compliant with IRB regulatory procedures transcribed the audio files. During the transcription process, any identifying information was redacted. Audio files were shared with the transcription service via a secure server. Quotes were linked to demographic factors via study IDs so participants’ privacy was protected to the best of our ability. Participants received a US $40 gift card for their time.

### Analysis

We computed descriptive statistics to characterize the sample by role (patients and caregivers). Content analysis was used to summarize the recommendations of the participants regarding the app design. Two researchers (KM and OC) initially reviewed and coded the transcripts independently using MAXQDA (VERBI Software GmbH) and then met to discuss the coding scheme. Themes were agreed upon and finalized by the researchers. Finally, we calculated descriptive frequencies for previous app use and the importance of features and content based on the Likert scale items using SPSS (version 32.0; IBM Corp).

## Results

### Participant Characteristics

Demographic and clinical characteristics of participants are listed in Table 1. All participants indicated that they had previously used a mobile app on their smartphone or tablet.

**Table 1:** Basic demographic and medical characteristics of patients and caregivers (N=20).

Variables		Patient (n=10)	Caregiver (n=10)
Sex: female, n (%)		4 (40)	9 (90)
Age (years), mean (range)		61.7 (45-74)	51.6 (24-71)
Race: White, n (%)		8 (80)	7 (70)
Ethnicity: Latino or Hispanic, n (%)		1 (10)	2 (20)
Spousal caregiver, n (%)		8 (80)	8 (80)
**Education, n (%)**			
	Some college or higher	7 (70)	10 (100)
	High school graduate	3 (30)	0 (0)
**Household income, n (%)**			
	<US $50,000	1 (10)	1 (10)
	≥US $50,000	6 (60)	7 (70)
	Decline to answer	3 (30)	2 (20)
**Employment status, n (%)**			
	Full-time	2 (20)	3 (30)
	Taking time off work for treatment or disability	4 (40)	2 (20)
	Unemployed, retired, or homemaker	4 (40)	5 (50)
**Primary disease site, n (%)**			
	Lung cancer	6 (60)	N/A^a^
	Esophageal cancer	4 (40)	N/A
**Stage at diagnosis, n (%)**			
	II	1 (10)	N/A
	III	9 (90)	N/A
**Eastern Cooperative Oncology Group performance status at recruitment, n (%)**			
	0	7 (70)	N/A
	1	3 (30)	N/A

^a^N/A: not applicable.

### Key Findings

Analysis of the data revealed insights into 4 key areas beneficial for app development. Participants valued the inclusion of *interactive elements*: 95% of participants preferred a live initial orientation with a yoga therapist who would conduct an assessment and provide recommendations for the level of practice (eg, “Having someone help someone be more comfortable and ease them into it, I think is always good,” said a female caregiver aged 24 years); 95% desired the yoga instructor’s contact information (“You cannot do away with the human contact. It’s a safety blanket,” said another female patient aged 56 years); and 90% showed interest in periodic check-ins to ensure engagement, with 65% preferring a weekly check-in (eg, “I like the once-a-week idea. Especially when you first start out,” expressed a caregiver aged 61 years; “100%. 100%. Yes, I think regular check-ins are crucial. I like a once-a-week check,” said a female patient aged 55 years).

Participants had varied preferences for the *instructional style* of the yoga sessions, with 45% favoring direct cueing by the instructor during the practice (eg, “It was easier to follow when she was just talking directly,” said a male patient aged 45 years), 35% of the participants opted for voice-over guidance (ie, narrator style; eg, “the voiceover might be a little bit better for me,” expressed a male patient aged 72 years), and 20% expressed no preference between direct cueing or voice-over guidance. Moreover, 45% preferred 1 video showing different instructors, each demonstrating a different level of practice difficulty (eg, “My husband and I have two different levels. I do the advanced and he do the moderate. We can see at the same time,” explained a female patient aged 56 years; “I think it makes it easier for the patient or the caregiver,” said a female patient aged 64 years), 45% preferred separate videos for each practice level (eg, “I would rather one single point to focus on. It was distracting. They were overlapping,” according to a male patient aged 59 years), and 10% expressed no preference regarding the demonstration of the different practice levels.

For content modules, participants highly favored segmented yoga practices with separate modules demonstrating mindful movements (ie, asanas), breathing exercises (ie, pranayama), and relaxation (ie, meditation; eg, “You may only have 15 minutes. You didn’t have a full hour. Just do the relaxation and your meditation. Practice just the breathing techniques,” said a male patient aged 58 years). They also found it essential to include detailed descriptions for each yoga video, such as the video length and a content breakdown.

Users emphasized a preference for a representative *yoga instructor*, with 80% of participants prioritizing instructors of various ages (eg, “Someone middle age or older. You wanted someone you can relate to. Someone a little bit more mature,” said a female patient aged 56 years; “I think different ages is nice. Especially if I’m over 60.” Expressed a female caregiver aged 61 years), 60% preferring instructors of various races and ethnicities (eg, “I think it’s more inclusive. People see themselves represented,” said a female caregiver aged 55 years), and 50% indicating a desire for instructors with diverse body types (eg, “I think ages would be really good, especially considering the demographic of the app. If somebody’s obese...that’s encouraging,” explained a female caregiver aged 24 years). Furthermore, 30% thought that the sex of the instructor was important (eg, “If you want the husbands to participate, they probably will want to see how a man actually performs some of the yoga,” said a male patient aged 63 years). Yet others thought that instructor characteristics were not important (eg, “I don’t think any of that matters as long as the person illustrating the technique is able to do it in a way that the observer, the trainee can understand it,” female caregiver aged 66 years).

Although over half (55%) of participants preferred a variety of instructors featured in the videos (“Three different instructors would be good,” said a male patient aged 71 years), others preferred just 1 instructor demonstrating all content (eg, “I would probably think the same instructor. You get used to 1 person or 1 person’s movements*,*” said a female patient aged 74 years). Additionally, 85% of participants found it important to know the instructor’s professional background, as it personalizes the app experience (eg, “I want to know about my instructor. It adds credibility,” explained a female patient aged 55 years).

Participants identified *enhancements* that should be included in the development of the app, with 95% of participants recommending session scheduling on a calendar (eg, “Something is scheduled and I’ve committed to it, it’s hard for me to cancel or not show up. It would encourage me to work towards consistency,” said a female caregiver aged 71 years), 95% desired a progress tracker that monitors session attendance (eg, “That way you know more or less how much you’re improving,” said a male patient aged 71 years), 90% wanted to receive practice reminders (eg, “Anything that will help,” said another male patient aged 72 years), and 90% preferred the provision of educational materials on how the yoga program can be used for symptom management (eg, “I think it’s important to know why you’re doing a particular exercise, what benefit to expect from the exercise,” expressed a female caregiver aged 66 years). Finally, 85% of participants wanted daily inspirational messages delivered via the app, with most of them preferring quotes and affirmations as well as participant testimonials (53%; eg, “Participants testimonials by all means. Any kind of motivating, inspiring quote you can get from cancer patients,” said a female patient aged 55 years).

## Discussion

The goal of this study was to inform the development of a mobile app to deliver a yoga program that was specifically designed for patients with thoracic cancer and their family caregivers. We interviewed patient-caregiver dyads with varying levels of recent yoga experience to solicit feedback on key design and content features following a user-centric approach. Our results provide insights into 4 key design areas, including interactive elements, instructional style, yoga instructor characteristics, and program enhancement features.

All participants indicated that developing a mobile yoga app would be important for patients and their families and that they would be interested in using such an app. At the same time, all participants desired at least some live interactions with a yoga instructor in the form of an initial live orientation, routine phone or video check-ins, and access to contact information for a yoga instructor in case of questions. Interestingly, the type and frequency of interactive components that were desired by our sample map onto the schedule of a previously efficacy-tested digital cardiopulmonary rehabilitation platform for surgical patients with lung cancer [16]. Guided by these findings, we will support our mobile yoga app with an instructor-led orientation session that includes an assessment of the patient’s and caregiver’s current fitness level followed by a recommendation of the yoga practice level to select on the app as a starting point (ie, chair-based, intermediate, or advanced practice). Moreover, as our app will be designed to support patients during active treatment, weekly check-ins may be beneficial, as participants may experience medical developments that require updating the initial practice recommendation and/or encouragement to adhere to the practice schedule.

Participants generally agreed on the importance of featuring instructors who are representative of the target audience regarding age, race, gender, or ethnicity, and body type (meaning inclusive of various body shapes and sizes). We believe this feedback is crucial, as commercially available yoga apps are limited in the diversity of yoga instructors, featuring almost exclusively White, young, lean women [23]. Moreover, most participants wanted to know about the instructors’ professional backgrounds, highlighting a desire for connection with the instructors.

In contrast, participants varied regarding their preferences for the instructional style, including the cueing of the practice and the demonstration of the various levels of practice (ie, separate videos per level vs 1 video demonstrating various levels). Because the original, instructor-led program was designed for dyads, and patients and caregivers may practice different modifications during their joint session, developing a video that demonstrates different levels of practice difficulty appears conducive to allowing family members to practice together. Moreover, in response to participants’ concerns, we will also create a separate video for participants who desire chair-based practice (ie, all exercises are performed while sitting in a chair). As such, we believe that we will accommodate participants’ feedback by developing 2 main variations of the yoga program. One version will be developed to demonstrate various levels of difficulty for participants who can stand and get on and off the ground, and another version will be specifically dedicated to chair practice (ie, the lowest intensity level).

Finally, participants identified motivational enhancements (scheduling, tracking, reminders, and inspiration) as important. Such features are common in mobile apps seeking to facilitate behavior change, and the prototype of our mobile yoga app will integrate these features [15,21].

Regarding next steps, we are currently conducting usability testing of the developed prototype of the mobile app via “think-out-loud” sessions. Once we refine the prototype, we will complete a single-arm trial to test intervention acceptability and identify an initial signal for intervention efficacy. Because both patients and caregivers desired human contact in the form of an orientation as well as intermittent touchpoints across the radiation treatment period, our digital intervention will involve 1 orientation session and weekly Zoom “check-ins.” We believe that integrating real-time instructor support will help ensure that patients not only engage with the app but also receive timely answers to their questions whenever they arise and ensure participant safety. Including caregivers in the study may address additional safety concerns for this self-administered yoga program. Because caregivers may be younger than patients, as seen in our sample (eg, adult children), they may also be more comfortable using technology and may be able to support the patient in navigating the app when necessary. In future work, it will be important to involve members of the clinical team in evaluating the yoga app to ensure its feasibility for integration into routine clinical care, identify potential implementation barriers early, and develop strategies to address these challenges.

Although these findings are novel and inform the development of a yoga app for patients and caregivers, our study includes noteworthy limitations. First, we used a convenience sample and recontacted participants who had already completed a symptom management intervention study. As such, our participants may have shown greater interest in the use of a yoga app to manage symptoms than the larger population. Moreover, participants had completed a “high touch” intervention and thus may desire instructor contact based on their prior experience. Thus, this sampling bias may limit the generalizability of our findings. Moreover, most participants had at least some prior mobile app experience, were relatively well educated, and were mostly non–Hispanic White. During the usability testing of the mobile yoga app, we will ensure enrollment of participants with diverse backgrounds, including those who are less familiar with mobile apps.

In summary, our sample of patients with thoracic cancers and their family caregivers provided relevant information in the areas of interactive elements, instructional style, yoga instructor characteristics, and program enhancement features. These findings will inform the translation of our current live yoga program to an on-demand mobile app. Our next steps will include field-testing the prototype using a single-arm trial design, with the long-term goal of increasing access to an evidence-based supportive care program in diverse clinical cancer care settings.

## Appendix

Multimedia Appendix 1 Mock-ups and interview guide.

## Abbreviations

ECOG: Eastern Cooperative Oncology Group
IRB: Institutional Review Board
MBC: Mindfulness Breast Care
QOL: quality of life

## Data Availability

The data generated during this study will be made available upon publication.

## References

[1] Lo SB, Ruprecht AL, Post KE, Eche-Ugwu IJ, Cooley ME, Temel JS, Greer JA (2024). Dyspnea-related dimensions and self-efficacy: associations with well-being in advanced lung cancer. J Pain Symptom Manage.

[2] Morrison EJ, Novotny PJ, Sloan JA, Yang P, Patten CA, Ruddy KJ, Clark MM (2017). Emotional problems, quality of life, and symptom burden in patients with lung cancer. Clin Lung Cancer.

[3] He Y, Sun LY, Peng KW, Luo MJ, Deng L, Tang T, You CX (2022). Sleep quality, anxiety and depression in advanced lung cancer: patients and caregivers. BMJ Support Palliat Care.

[4] Seo YJ, Park H (2019). Factors influencing caregiver burden in families of hospitalised patients with lung cancer. J Clin Nurs.

[5] Porter LS, Keefe FJ, Garst J, McBride CM, Baucom D (2008). Self-efficacy for managing pain, symptoms, and function in patients with lung cancer and their informal caregivers: associations with symptoms and distress. Pain.

[6] Ding Z, Fan Y, Zhong G, Zhang X, Li X, Qiao Y, Cui H (2024). A dyadic analysis of family adaptation among breast cancer patients and their spouses based on the framework of family stress coping theory. Front Public Health.

[7] Liu W, Lewis FM, Oxford M, Kantrowitz-Gordon I (2024). Common dyadic coping and its congruence in couples facing breast cancer: the impact on couples' psychological distress. Psychooncology.

[8] Lu H, Ma RC, Yin YY, Song CY, Yang TT, Xie J (2023). Clinical indicators of effects of yoga breathing exercises on patients with lung cancer after surgical resection: a randomized controlled trial. Cancer Nurs (Forthcoming).

[9] Milbury K, Liao Z, Shannon V, Mallaiah S, Nagarathna R, Li Y, Yang C, Carmack C, Bruera E, Cohen L (2019). Dyadic yoga program for patients undergoing thoracic radiotherapy and their family caregivers: results of a pilot randomized controlled trial. Psychooncology.

[10] Milbury K, Mallaiah S, Lopez G, Liao Z, Yang C, Carmack C, Chaoul A, Spelman A, Cohen L (2015). Vivekananda yoga program for patients with advanced lung cancer and their family caregivers. Integr Cancer Ther.

[11] Milbury K, Chen A, Li Y, Liao Z, Whisenant M, Mama SK, Bentley JR, Shannon V, Yousuf S, Silva RF, Tsao AS, Bruera E, Cohen L (2026). Yoga for patients undergoing thoracic radiotherapy and their family caregivers: results of a randomized controlled trial. JCO Oncol Pract (Forthcoming).

[12] Sharma S, Gergen Barnett K, Maypole JJ, Grochow Mishuris R (2022). Evaluation of mHealth apps for diverse, low-income patient populations: framework development and application study. JMIR Form Res.

[13] Sereno M, Iniesta-Chamorro JM, Garrido-Rubiales B, Gomez EJ, Casado Sáenz E (2023). M-health in lung cancer: a literature review. SAGE Open Med.

[14] Buneviciene I, Mekary RA, Smith TR, Onnela JP, Bunevicius A (2021). Can mHealth interventions improve quality of life of cancer patients? A systematic review and meta-analysis. Crit Rev Oncol Hematol.

[15] Henshall C, Davey Z (2020). Development of an app for lung cancer survivors (iEXHALE) to increase exercise activity and improve symptoms of fatigue, breathlessness and depression. Psychooncology.

[16] Li G, Zhou X, Deng J, Wang J, Ai P, Zeng J, Ma X, Liao H (2025). Digital therapeutics-based cardio-oncology rehabilitation for lung cancer survivors: randomized controlled trial. JMIR Mhealth Uhealth.

[17] Park S, Kim JY, Lee JC, Kim HR, Song S, Kwon H, Ji W, Choi CM (2019). Mobile phone app-based pulmonary rehabilitation for chemotherapy-treated patients with advanced lung cancer: pilot study. JMIR Mhealth Uhealth.

[18] Ji W, Kwon H, Lee S, Kim S, Hong JS, Park YR, Kim HR, Lee JC, Jung EJ, Kim D, Choi CM (2019). Mobile health management platform-based pulmonary rehabilitation for patients with non-small cell lung cancer: prospective clinical trial. JMIR Mhealth Uhealth.

[19] Huberty J, Bhuiyan N, Puzia M, Joeman L, Larkey L, Mesa R (2022). Meditation mobile app developed for patients with and survivors of cancer: feasibility randomized controlled trial. JMIR Cancer.

[20] Lin H, Zhong Y, Zheng S, Jiang X, Xu Y, Chan S, Liu S, Chow KM, Yang Q, Huang W, Zhu J (2026). Effectiveness and cost-effectiveness of an app-based mindfulness breast care program for reducing body image distress and stigma among survivors of breast cancer: randomized controlled trial. J Med Internet Res.

[21] Zheng S, Huang W, Zhang X, Hua Y, Chan S, Liu S, Zhong Y, Jiang X, Zhu J (2025). A Mindfulness Breast Care App to reduce body image distress and stigma for breast cancer survivors: development and preliminary quality assessment. Int J Nurs Sci.

[22] Guest G, Bunce A, Johnson L (2006). How many interviews are enough?: An experiment with data saturation and variability. Field Methods.

[23] Webb JB, Vinoski ER, Warren-Findlow J, Padro MP, Burris EN, Suddreth EM (2017). Is the "yoga bod" the new skinny?: A comparative content analysis of mainstream yoga lifestyle magazine covers. Body Image.

