# Supplementary material for: A User-Centric Approach to the Development of a Mobile Yoga App for Patients With Thoracic Cancer and Their Family Caregivers
Source: JMIR Form Res. 2026 Oct 30;10:e89649. doi: 10.2196/89649 (PMC13768818; doi:10.2196/89649)
Supplement: Supplementary file 1 [file formative_v10i1e89649_app1.pdf]

THE UNIVERSITY OF TEXAS
MD Anderson Cancer Center
Making Cancer History®

Interview Guide and Mockups to Inform the Development of a Yoga App

8/2/2026 1

1. Do you currently use any mobile apps on your phone or tables?

a) If yes, what types of apps do you use and please ell me about any experiences you have had using an app for exercise previously.

b) If no, are there any specific reasons?

2. How would you feel about participating in a yoga program using a mobile application on your phone or tablet?

<u>Optional probe questions</u>
a) How comfortable would you feel using an app on your phone or tablet for yoga?
b) What do you think would be challenging when using an app on your phone or tablet for yoga?
c) What are some benefits of using an app on your phone or tablet for yoga?

3. The first session will be live (via zoom) with the yoga therapist who will do an assessment and recommendations of the level of practice. Is this important for you?
( ) Yes ( ) No

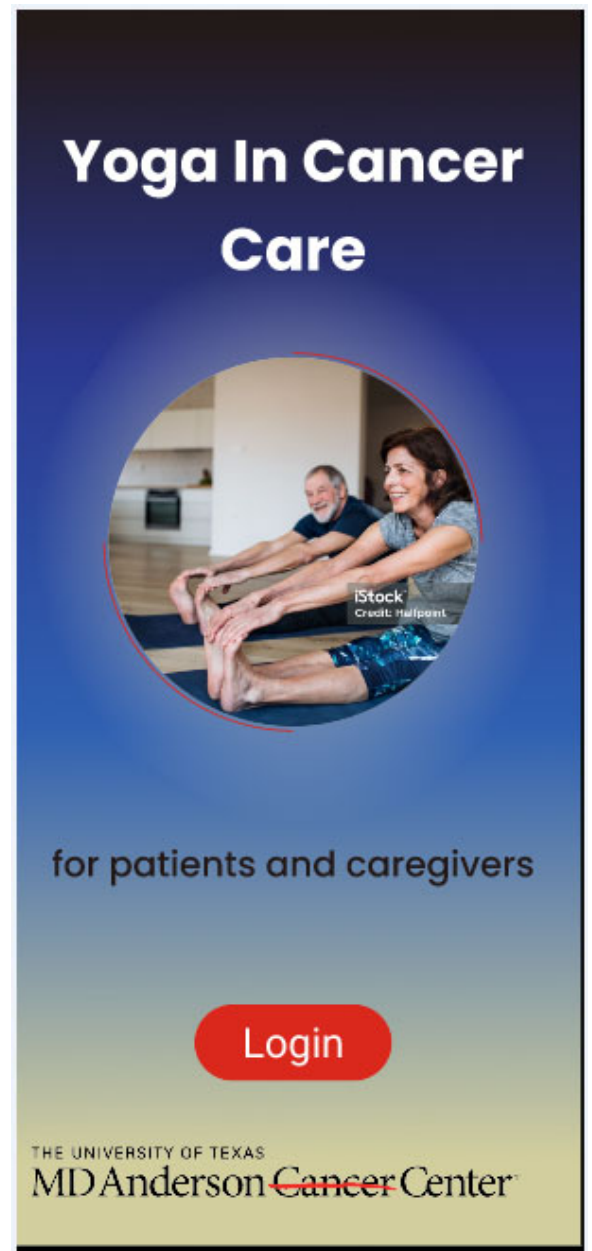

8/2/2026 2

1. What do you think about this first screen.

2. Do you like the picture?
( ) Yes ( ) No What would you like to see here?

3. Do you like the name of the program?
( ) Yes ( ) No

If not what about other names: ( ) Yoga in Oncology Care, ( ) Yoga for Cancer, ( ) Yoga and Cancer, ( ) other

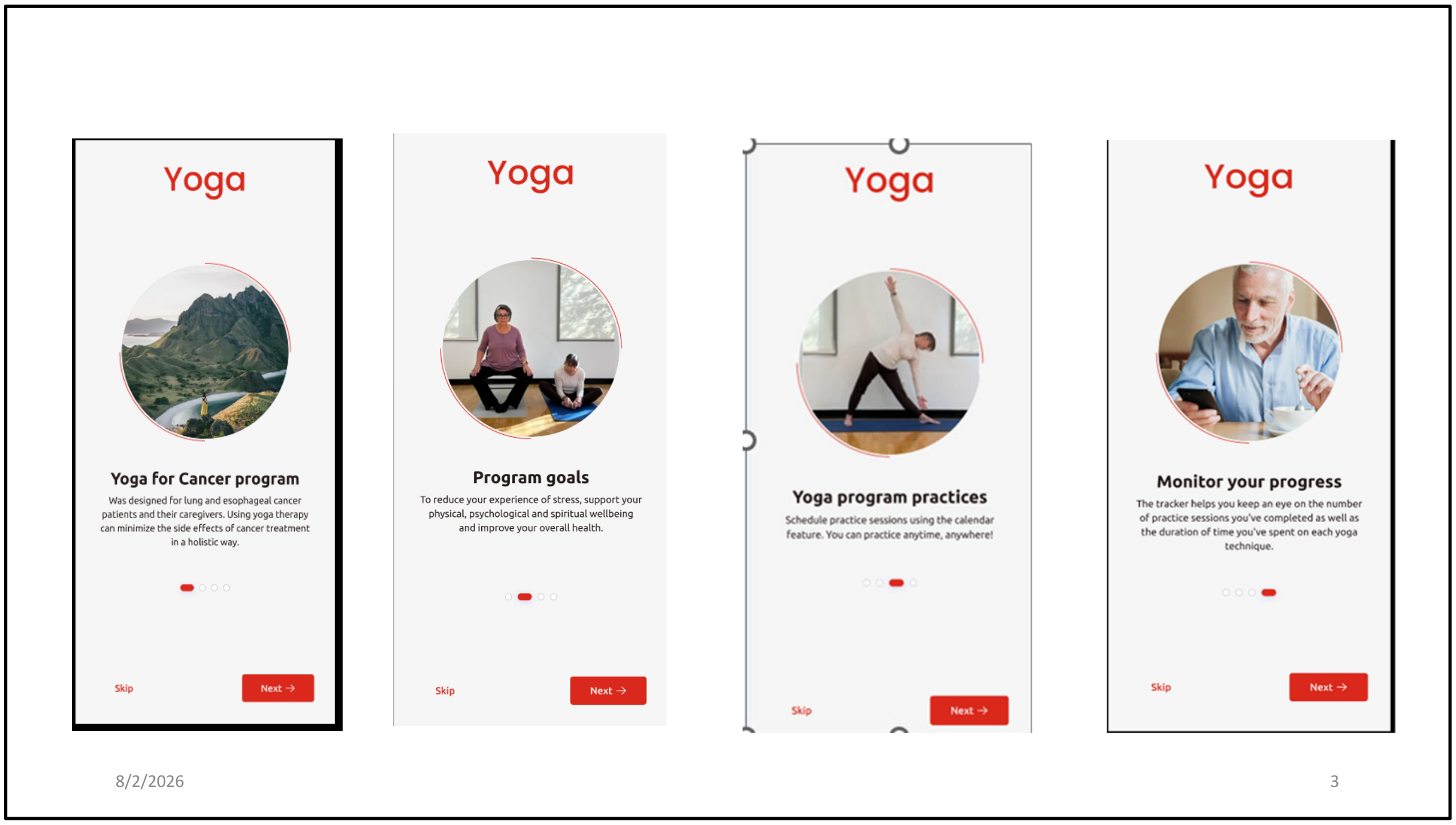

The first time you enter the app, after creating your login, you will be shown these screens with the program information, goal, practices and progress.

1. Do you think it is important to have these information?
( ) Yes ( ) No
If yes, would you like to have:
( ) More Information ( ) Less Information ( ) The actual information is enough.

3. Do you like the pictures?
( ) Yes ( ) No
If not, what would you like to see here?____________________

4. Do you have any other suggestions?

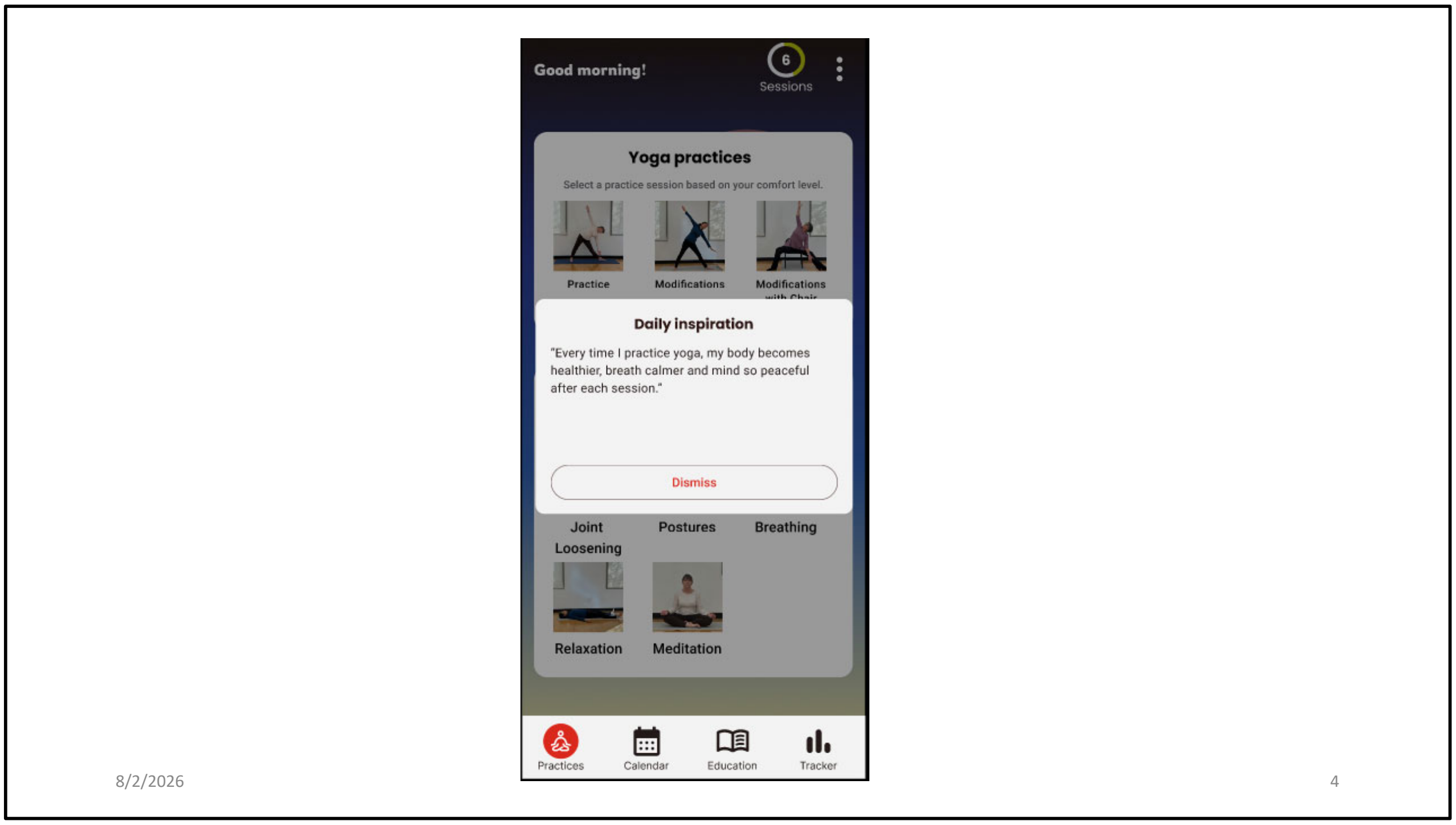

After you login you will see this screen with inspirations quotes.

1. Do you think it is important to have the inspirations quotes?
( ) Yes ( ) No
If Yes, Do you have preference for the inspiration quotes:
( ) random quotes and affirmations
( ) participants testimonials
( ) both

3. How frequently would you like the quote to appear in your screen?
( ) every login
( ) first login of the day
( ) first login of the week
( ) Other _________________

4. Do you have any other suggestions?

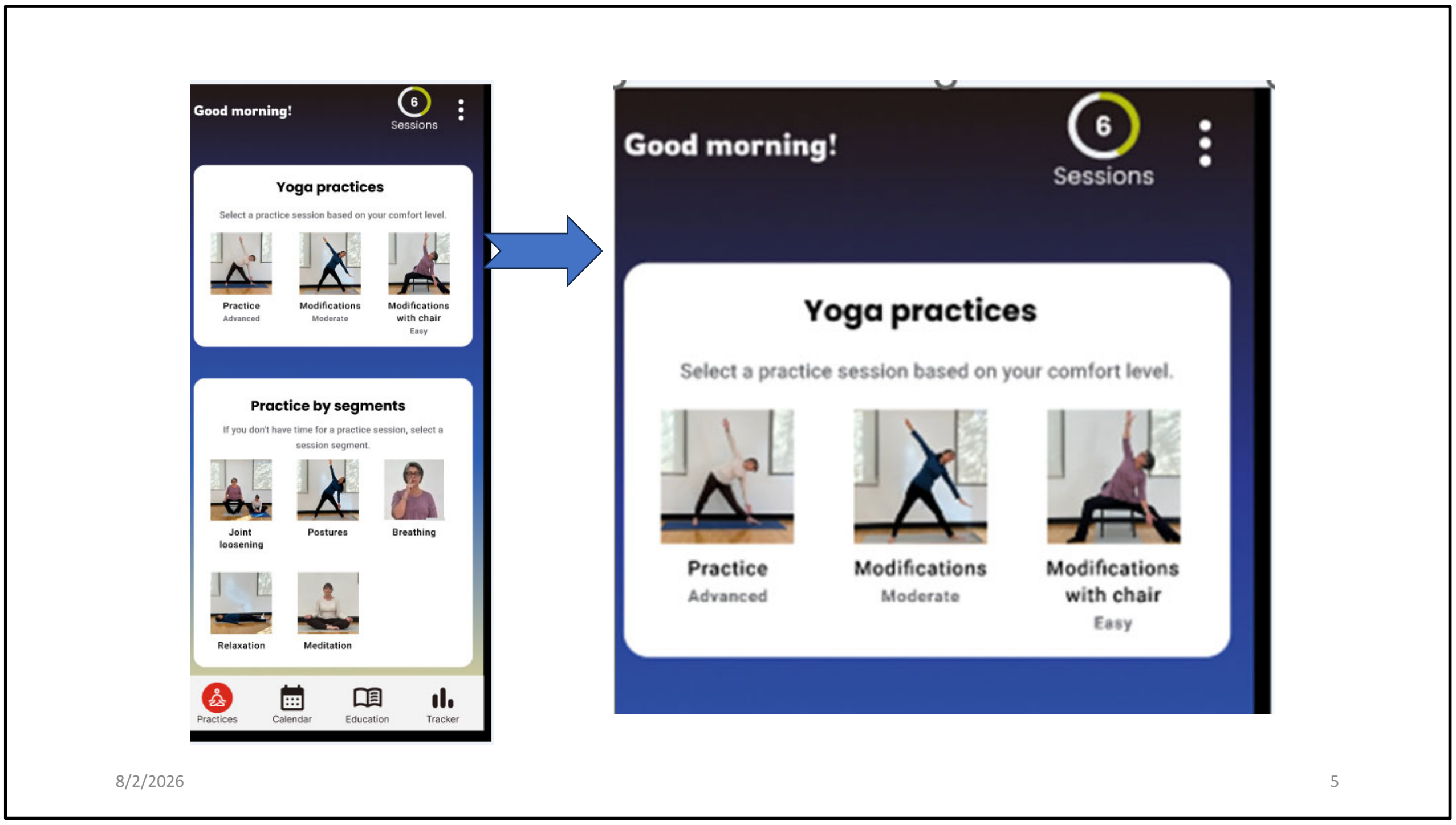

1. In the Instructor representation, how important are the following?

- **Gender/sex:** ( ) Important: Preference____________ ( )Not Important ( ) Neutral
- **Age:** ( ) Important: Preference____________ ( )Not Important ( ) Neutral
- **Body type:** ( ) Important: Preference____________ ( )Not Important ( ) Neutral
- **Ethnicity:** ( ) Important: Preference____________ ( )Not Important ( ) Neutral

If important, 'Tell me more about that' and/or 'Do you have specific preferences regarding.

2. Would you prefer to have all the pictures of?
( ) same instructor
( ) different instructors
( ) no preference

3. How do you feel about having three options? (advance, moderate and easy). Any other suggestions to describe the practice levels?

5. Do you like the greetings on top left?
( ) Yes ( ) No ( ) other

6. Do you like to have the sessions track on the top right?
( ) Yes ( ) No ( ) other

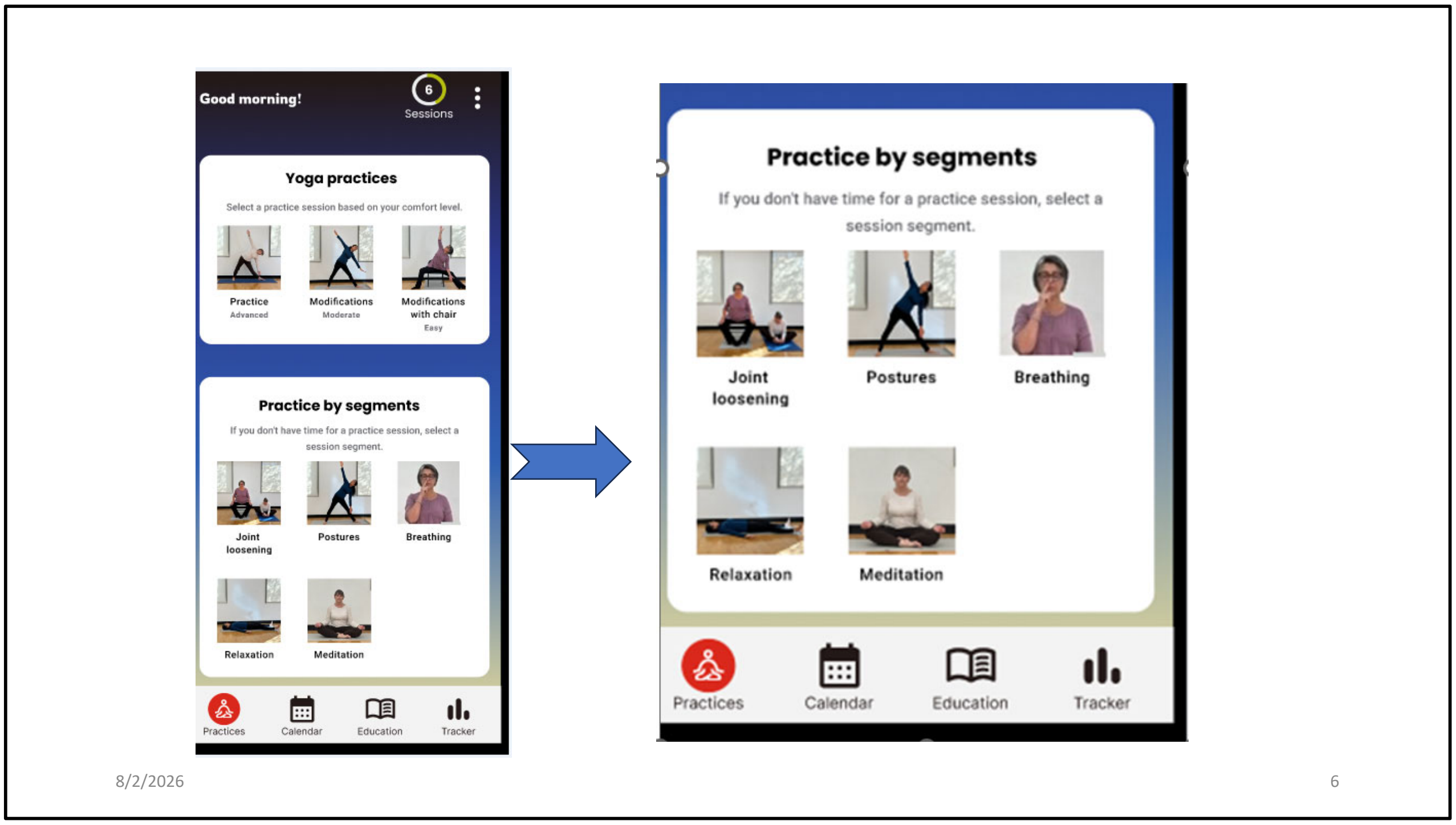

1. Do you like to have the options to practice by segment?
( ) Yes ( ) No

2. Would you prefer to have all the pictures of?
( ) same instructor
( ) different instructors
( ) no preference

3. Do you have any other suggestions?

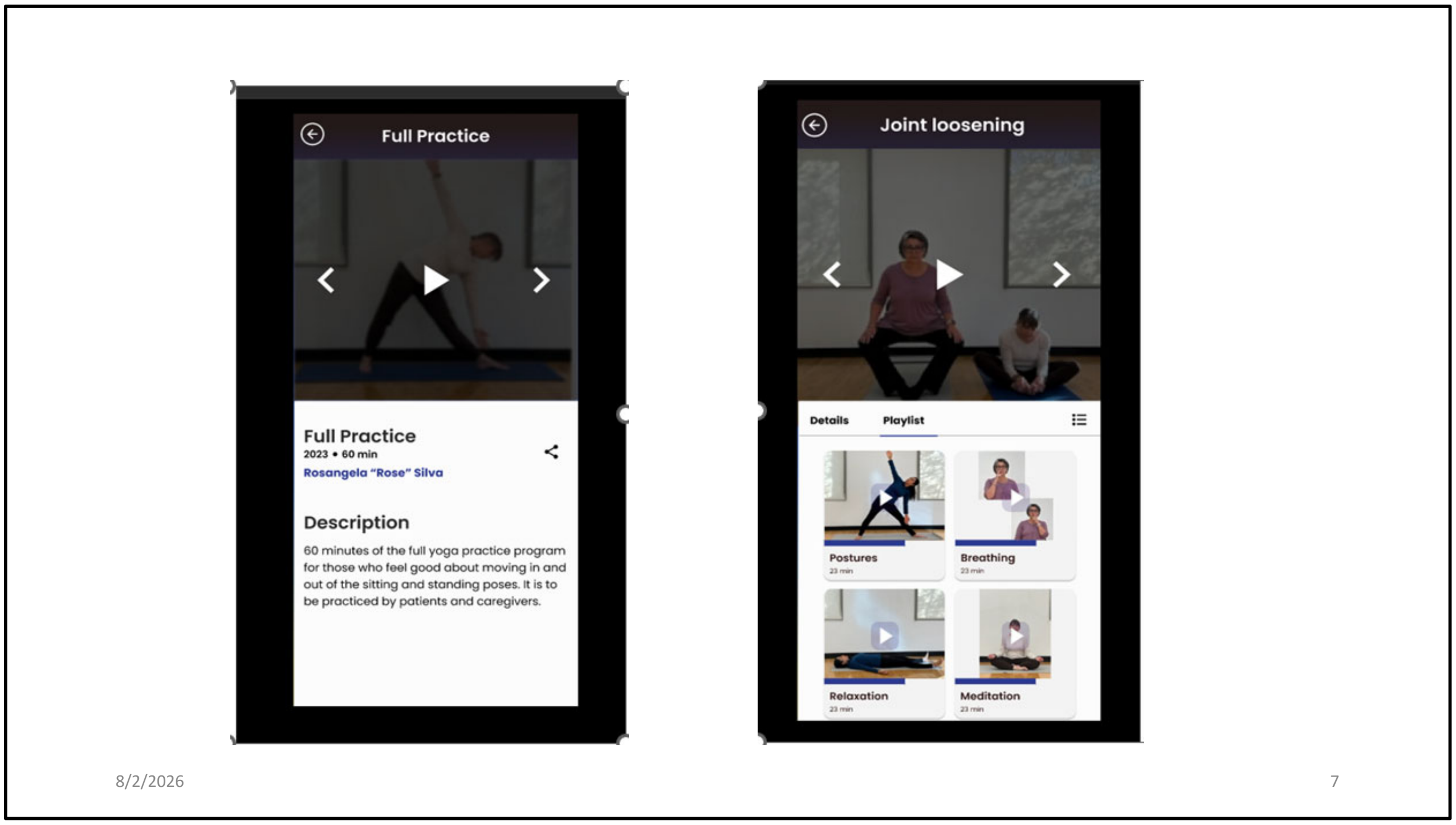

1. What do you think about the videos?

2. What is your video demonstration preference?
( ) prefer one video with 3 levels?
( ) prefer three different videos, one for each level.
( ) no preference

3. Do you think it is important to have the description of the practice information?
( ) Yes ( ) No If yes, would you like to have: ( ) More Information ( ) Less Information ( ) The actual information is enough.

4. Do you think this format will be easy to follow by someone that has not practiced yoga before?
( ) Yes ( ) No

5. Do you have any other suggestions?

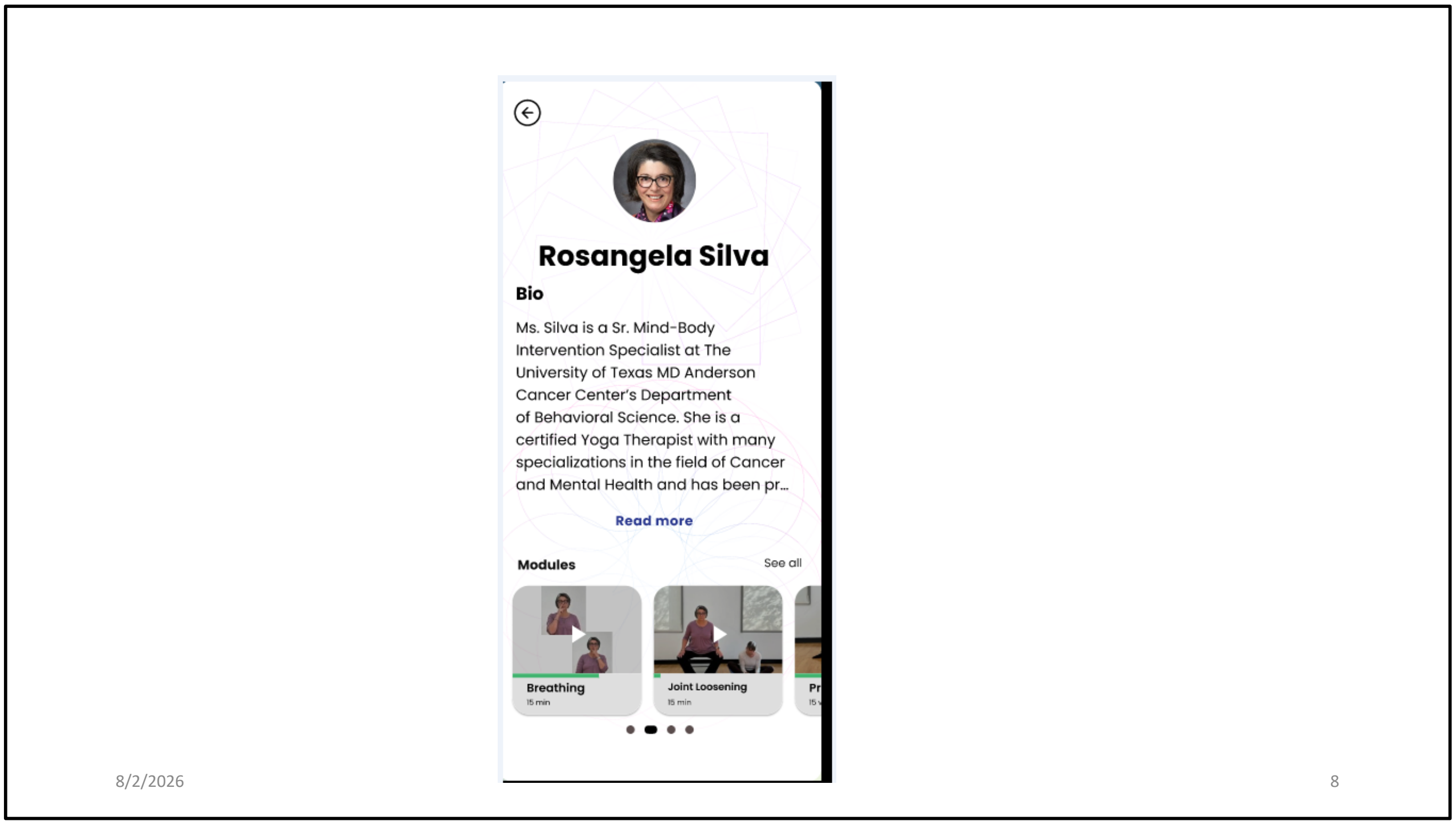

1. Do you think it is important to know the instructors background?
( ) Yes ( ) No

2. Do you have any other suggestions?

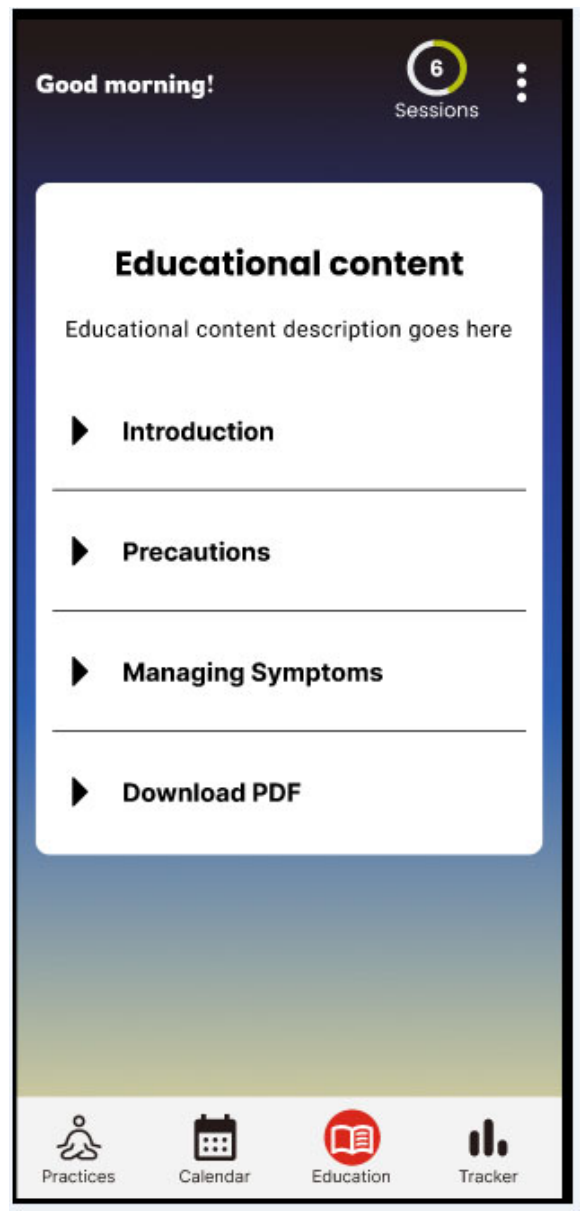

8/2/2026 9

1. Do you think it is important to have the Yoga Manual to be accessed by the app?
( ) Yes ( ) No

2. Do you think it is important to have access to the manual by topics?
( ) Yes ( ) No

3. Do you have any other suggestions?

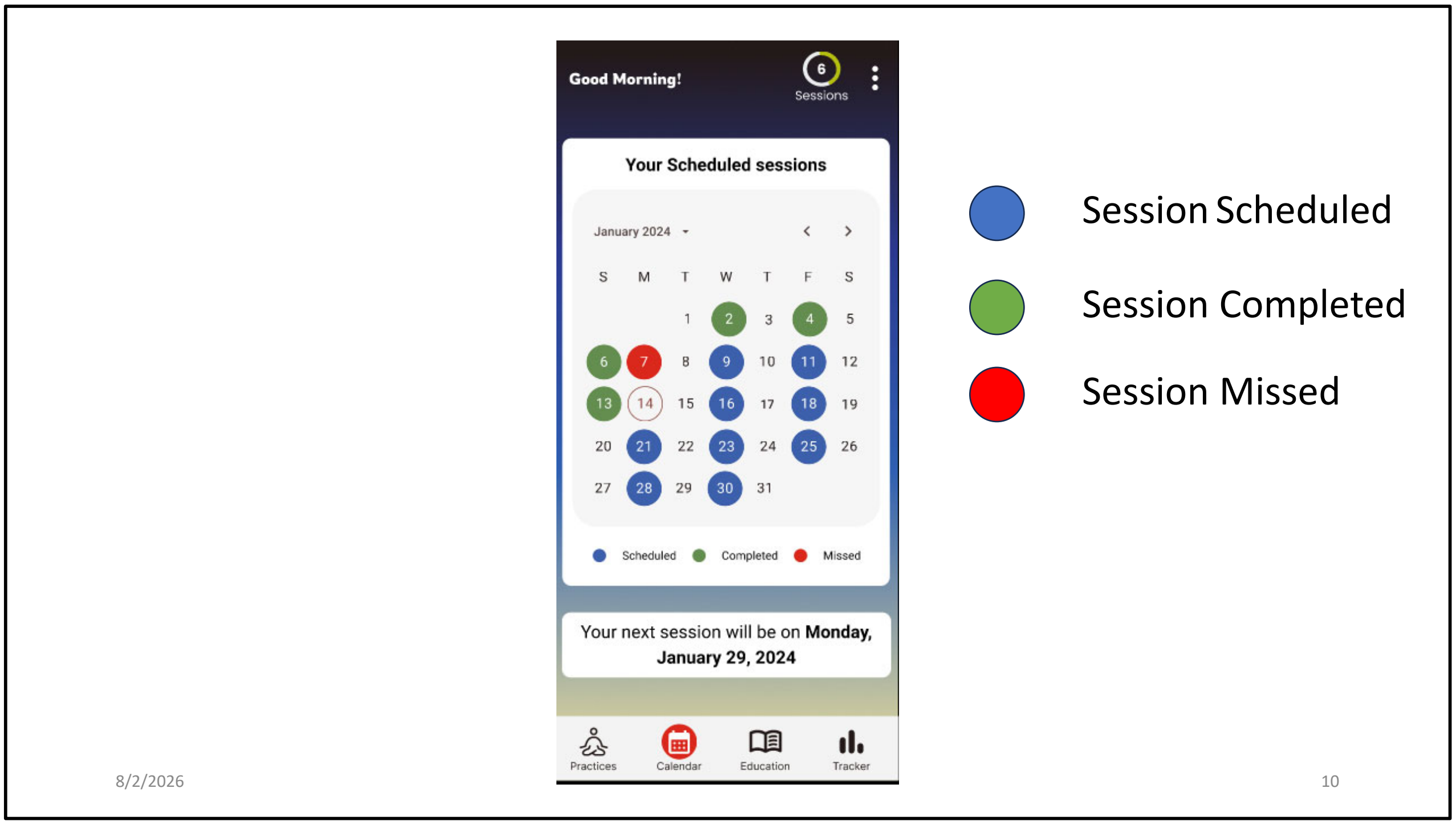

1. Do you think it is important to have the sessions pre-scheduled during the first assessment session with the Instructor?
( ) Yes ( ) No

2. What do you think about the schedule legend?
( ) Yes ( ) No

3. Do you like the colors representation?
( ) Yes ( ) No

4. Would you like to receive reminders of the sessions?
( ) the day before ( ) the day off ( ) 1 hour prior ( ) fifteen minutes prior

5. Do you like to have a reminder for the next session on the bottom?
( ) Yes ( ) No

6. Would you like the option to change/schedule and re-schedule the sessions yourself?
( ) Yes ( ) No

7. How do you feel about the reminder about the next session on the bottom?

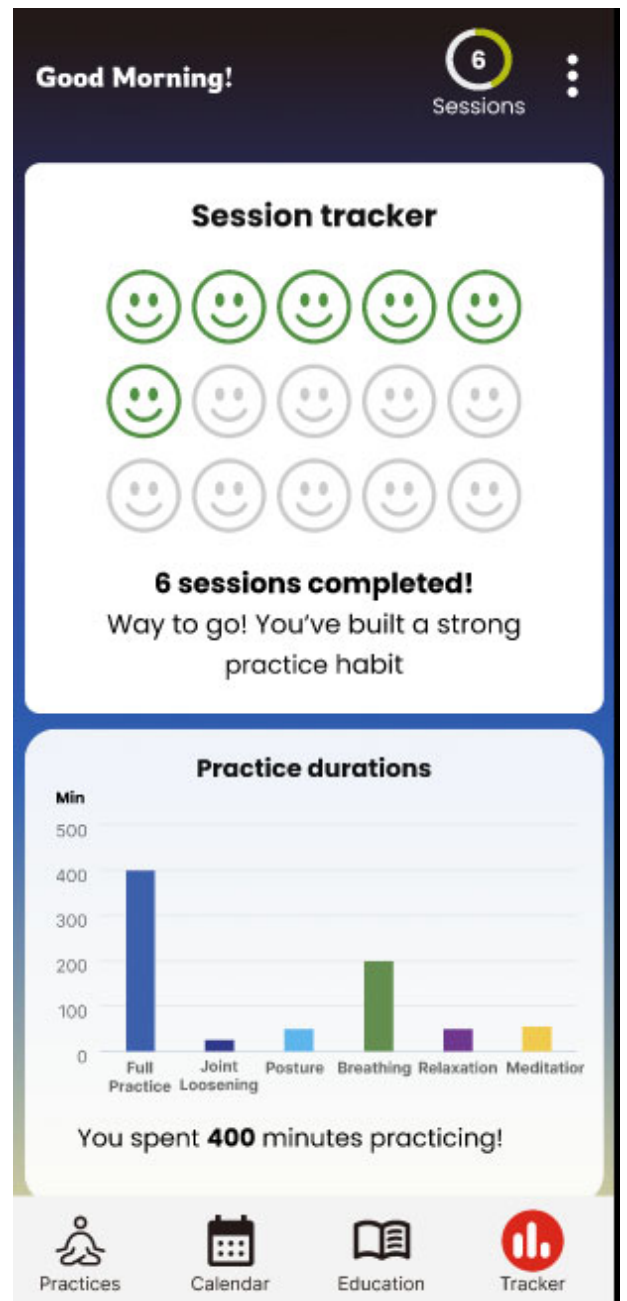

8/2/2026

11

1. Do you think it is important to have the emoji showing session progress?

( ) Yes ( ) No ( ) Neutral ( ) Other

2. What do you think about the graphic on the bottom showing minutes of sessions completed?

3. Any other suggestion?

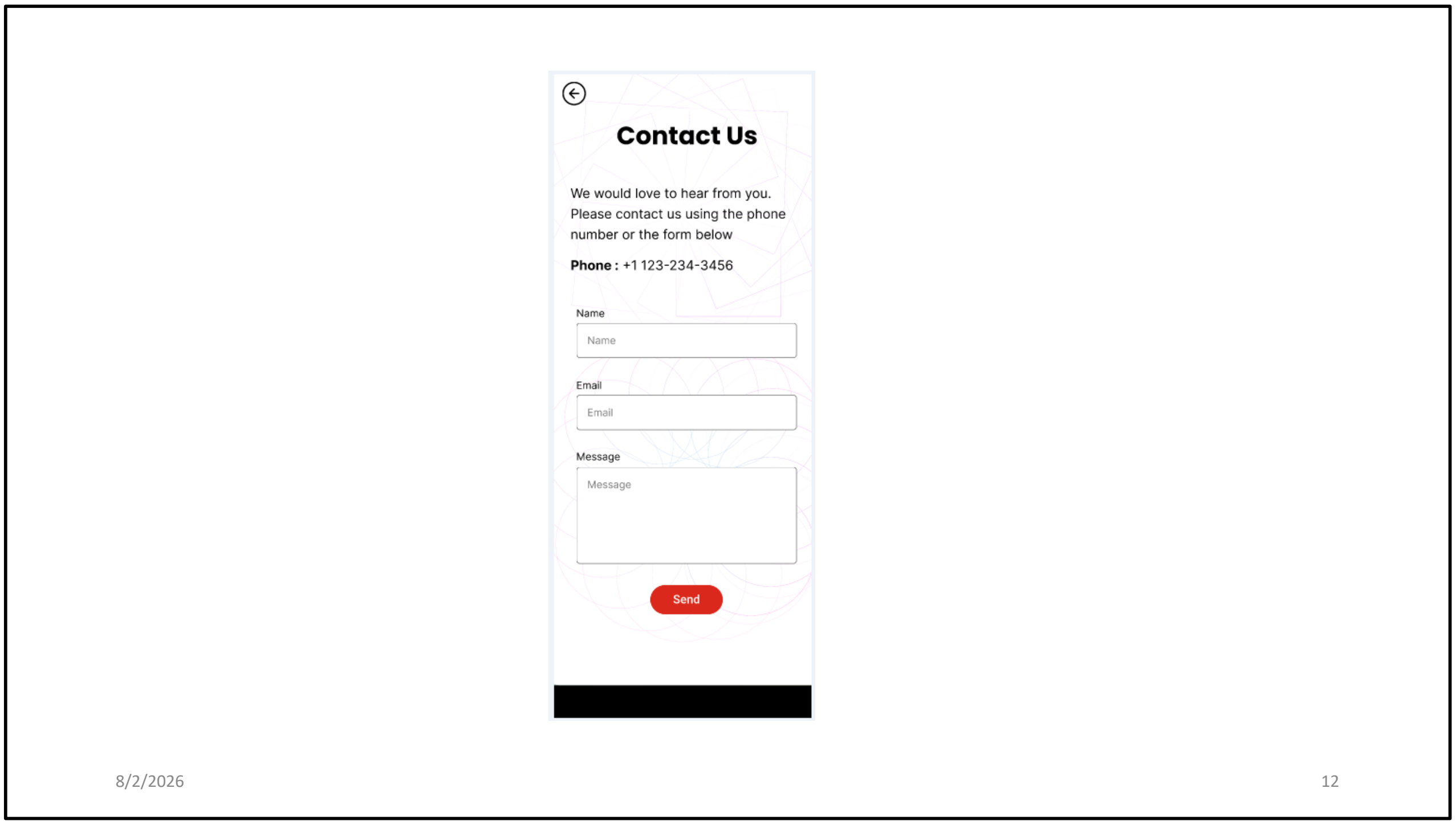

1. Do you think it is important to have the instructor information contact?
( ) Yes ( ) No

2. What are things you envision using the contact information for?

3. Do you have other suggestions?

Thank you! This concludes our interview.
